# Time Course of Asymptomatic Stenosis in Multiple Lumbar Spinal Stenosis—Five-Year Results of Selective Decompression of Symptomatic Levels

**DOI:** 10.3390/medicina60040636

**Published:** 2024-04-15

**Authors:** Kazuyuki Watanabe, Koji Otani, Takuya Nikaido, Kinshi Kato, Hiroshi Kobayashi, Shoji Yabuki, Shin-ichi Konno, Yoshihiro Matsumoto

**Affiliations:** Department of Orthopaedic Surgery, Fukushima Medical University School of Medicine, 1 Hikarigaoka, Fukushima 960-1295, Japan; kotani@fmu.ac.jp (K.O.); tnikaido@fmu.ac.jp (T.N.); kinshi@fmu.ac.jp (K.K.); hiroshik@fmu.ac.jp (H.K.); yabuki@fmu.ac.jp (S.Y.); ymatsu@fmu.ac.jp (Y.M.)

**Keywords:** lumbar spinal stenosis, asymptomatic stenosis, surgical outcome, decompression, dural sac cross-sectional area, natural course, neurological finding, gait-loading test, lumbar extension-loading test, prophylactic decompression

## Abstract

*Background*: In the diagnosis of lumbar spinal stenosis (LSS), finding stenosis with magnetic resonance imaging (MRI) does not always correlate with symptoms such as sciatica or intermittent claudication. We perform decompression surgery only for cases where the levels diagnosed from neurological findings are symptomatic, even if multiple stenoses are observed on MRI. The objective of this study was to examine the time course of asymptomatic stenosis in patients with LSS after they underwent decompression surgery for symptomatic stenosis. *Materials and Methods*: The participants in this study comprised 137 LSS patients who underwent single-level L4–5 decompression surgery from 2003 to 2013. The dural sac cross-sectional area at the L3–4 disc level was calculated based on preoperative MRI. A cross-sectional area less than 50 mm^2^ was defined as stenosis. The patients were grouped, according to additional spinal stenosis at the L3–4 level, into a double group (16 cases) with L3–4 stenosis, and a single group (121 cases) without L3–4 stenosis. Incidences of new-onset symptoms originating from L3–4 and additional L3–4-level surgery were examined. *Results*: Five years after surgery, 98 cases (72%) completed follow-up. During follow-up, 2 of 12 patients in the double group (16.7%) and 9 of 86 patients in the single group (10.5%) presented with new-onset symptoms originating from L3–4, showing no significant difference between groups. Additional L3–4 surgery was performed for one patient (8.3%) in the double group and three patients (3.5%) in the single group; again, no significant difference was shown. *Conclusion*: Patients with asymptomatic L3–4 stenosis on preoperative MRI were not prone to develop new symptoms or need additional L3–4-level surgery within 5 years after surgery when compared to patients without preoperative L3–4 stenosis. These results indicate that prophylactic decompression for asymptomatic levels is unnecessary.

## 1. Introduction

Lumbar spinal stenosis (LSS) characteristically causes neurogenic intermittent claudication, radicular pain, and sensory and motor disturbances in the lower extremities [1]. In the diagnosis of LSS, magnetic resonance imaging (MRI) represents one of the most practical examinations, allowing non-invasive observation of the spinal canal. In the treatment of LSS, surgery is usually performed when conservative treatment proves ineffective, or when neurological symptoms such as bowel/bladder dysfunction and lower extremities paralyses are severe. A wide variety of surgical procedures are available for LSS, and the basic surgical technique is laminectomy, which involves posterior decompression of the stenotic level. In addition to the conventional technique of decompression with dissection of paraspinal muscles on both sides, less invasive procedures using various endoscopic and microscopic techniques have been reported [2,3]. Usually, decompression with any technique is considered for stenotic levels that are apparent on MRI. However, findings of spinal stenosis (dural sac compression) on MRI do not always relate to clinical symptoms. Healthy individuals often show spinal stenosis and other degenerative findings on MRI without clinical symptoms [4,5,6]. Some studies have also shown no correlation between symptom severity and the cross-sectional area of the spinal canal in LSS patients [7,8,9,10]. Thus, diagnosing symptomatic stenosis levels based on MRI alone is inappropriate, and performing decompression surgery for every level of dural sac compression seen on MRI may represent over-treatment if areas of asymptomatic stenosis are present. One previous study showed that when double-level stenosis was observed on radiological examination, the symptomatic level was usually one level, with the other being asymptomatic [11]. We should avoid performing unnecessary decompressions as much as possible to decrease operative time, intraoperative bleeding, and surgical invasiveness. As for complications, multilevel decompression has been reported as a risk factor for symptomatic epidural hematoma [12]. Further, wide-ranging decompression is associated with a higher risk of adhesion of the cauda equina [13].

In LSS patients showing multiple-level stenoses on MRI, a truly symptomatic level should be diagnosed from neurological findings. In addition to examination during a resting state, neurological evaluation based on the gait-loading test [11,14] and lumbar extension-loading test [15]; functional diagnoses based on selective nerve root block [11] are performed to determine the symptomatic level responsible for lumbosacral symptoms. In our institution, we apply decompression surgery only for symptomatic levels that are diagnosed using neurological findings from the above evaluations, even if stenosis is evident at other levels on MRI. However, no previous studies appear to have examined the long-term postoperative course of asymptomatic stenosis, and whether untreated asymptomatic stenosis affects surgical outcomes of decompression surgery for LSS. The purpose of this study was to reveal whether asymptomatic stenosis induces symptoms, and to clarify the influence of untreated asymptomatic stenosis on outcomes following decompression surgeries that were limited to symptomatic levels.

## 2. Materials and Methods

This retrospective cohort study used a database to prospectively record the outcomes of spinal surgeries and medical records in our institution. The study protocol was approved by the ethics committee of our institution. Consecutive LSS patients who underwent conventional or microendoscopic single-level L4–5 decompression surgery from 2003 to 2013 were included in this study. Postoperatively, the patients were allowed to walk the day after surgery, and were usually discharged within one to two weeks without specialized rehabilitation. Cases with foraminal stenosis, lumbosacral transitional vertebrae, previous spinal surgery, revision surgery, or any instrumentation or fusion surgery were excluded from this study. Patients with a history of rheumatoid arthritis, cervical or thoracic spinal disease, or destructive spondyloarthropathy were likewise excluded. The patients were divided into two groups and compared according to the presence of L3–4 stenosis on preoperative MRI (see below). Follow-up was continued for 5 years. Patients were followed up at the outpatient clinic, once a year after surgery. If a patient experienced any problems, such as recurrence of pain, they could visit our clinic. The primary outcome was a new onset of symptoms originating from L3–4 stenosis during follow-up. Anterior thigh pain and numbness were considered symptoms from L3–4 stenosis (L4 radiculopathy), and were confirmed by neurological findings and L4 nerve root block. Secondary outcomes were additional surgery for L3–4 stenosis, L5 residual symptoms (any leg pain or numbness in the same area as before surgery), and revision L4–5-level surgery within 5 years. The numerical rating scale (NRS) scores for low back pain, leg pain, leg numbness, and satisfaction for surgery (0, unsatisfied; 10, completely satisfied), as well as the Roland–Morris Disability Questionnaire (RDQ) [16] results were also examined as secondary outcomes at the 5-year follow-up. In addition to the total RDQ score, the norm-based RDQ score has been established for the Japanese version of RDQ [17]. The norm-based RDQ score was calculated using the mean RDQ (baseline) and standard deviation, based on the sex and age of complainants with low back pain from a national survey in Japan [17,18]. The national norm was converted to 50 points and the standard deviation to 10. Norm-based RDQ scores >50 indicate better low back pain-related quality of life than the national average, and <50 indicates a worse low back pain-related quality of life than the national average. Norm-based RDQ scores can be evaluated by adjusting for the effects of aging for the follow-up period. Age, sex, body mass index (BMI), smoking, diabetes mellitus (DM), L4 spondylolisthesis (Mayerding grade I or more), and use of microendoscope were also compared.

### 2.1. Definition of L3–4 Stenosis

The dural sac cross-sectional area (DSCA) at the L3–4 disc level was calculated using preoperative MRI. T2-weighted axial views of the L3–4 disc level were assessed using imaging software (Image J, 1.53e; National Institutes of Health, Bethesda, MD, USA) by a single board-certificated spine surgeon blinded to other patient information. A cross-sectional area <50 mm^2^ was defined as stenosis, according to a previous study [19].

### 2.2. Diagnosis of Symptomatic and Asymptomatic Levels

We diagnosed symptomatic pathological levels with multiple-level stenoses on MRI according to the neurological findings presented in Table 1 [11,15,20,21]. Motor function, sensory function, and deep tendon reflexes were first examined at rest. Then, a gait-loading test [11,14] and a lumbar extension-loading test [15] were conducted, and the neurological findings were examined again. In the gait-loading test, we instructed the patient to walk at their average speed and keep their back straight for as long as possible [11]. Changes in the degree and area of symptoms were recorded during the loading test. Patients with neurogenic intermittent claudication usually reported that their symptoms worsened under loading, and stopped walking because of pain and numbness. Just after stopping, the neurological findings were quickly examined before the symptoms resolved. If the symptoms did not extend to the anterior thigh and the patient showed a normal patellar tendon reflex (PTR), normal strength of the quadriceps and iliopsoas muscles, and no sensory disturbance in the L4 area (medial side of the knee) not only in the rest state, but also immediately after gait loading and extension loading, we considered the L4 nerve root to be unaffected.

### 2.3. Statistical Analysis

The Mann–Whitney *U* test was performed for continuous variables, and the chi-square test was applied for comparisons involving categorical variables. For analyzing changes in symptoms and RDQ after surgery, the paired *t*-test was used to compare NRS and RDQ scores preoperatively and 5 years postoperatively. All statistical analyses were performed using JMP software (version 15.0; SAS Institute, Cary, NC, USA). Values of *p* < 0.05 were considered significant.

## 3. Results

### 3.1. Recruitment of Patients

**Figure 1:**
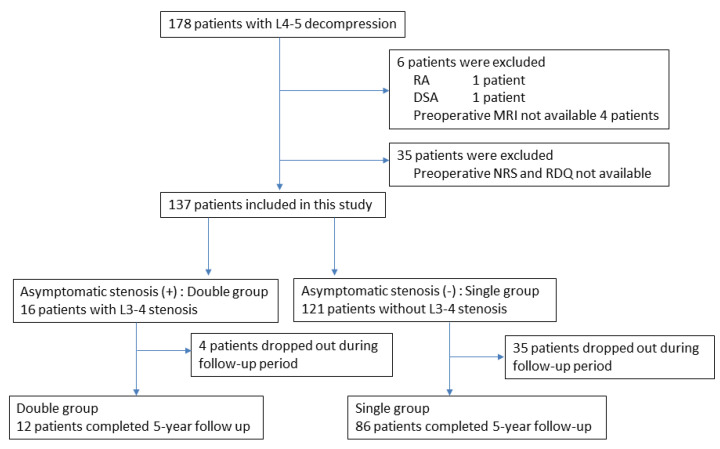
Flowchart of patient recruitment. Of the 178 patients who underwent L4–5 decompression surgery, 137 (77%) were included in this study. These included 16 patients with asymptomatic stenosis in addition to symptomatic L4–5 stenosis (double group), and 121 patients with only symptomatic L4–5 stenosis (single group). Of these, 12 patients in the double group and 86 patients in the single group completed 5 years of follow-up.

**Figure 2:**
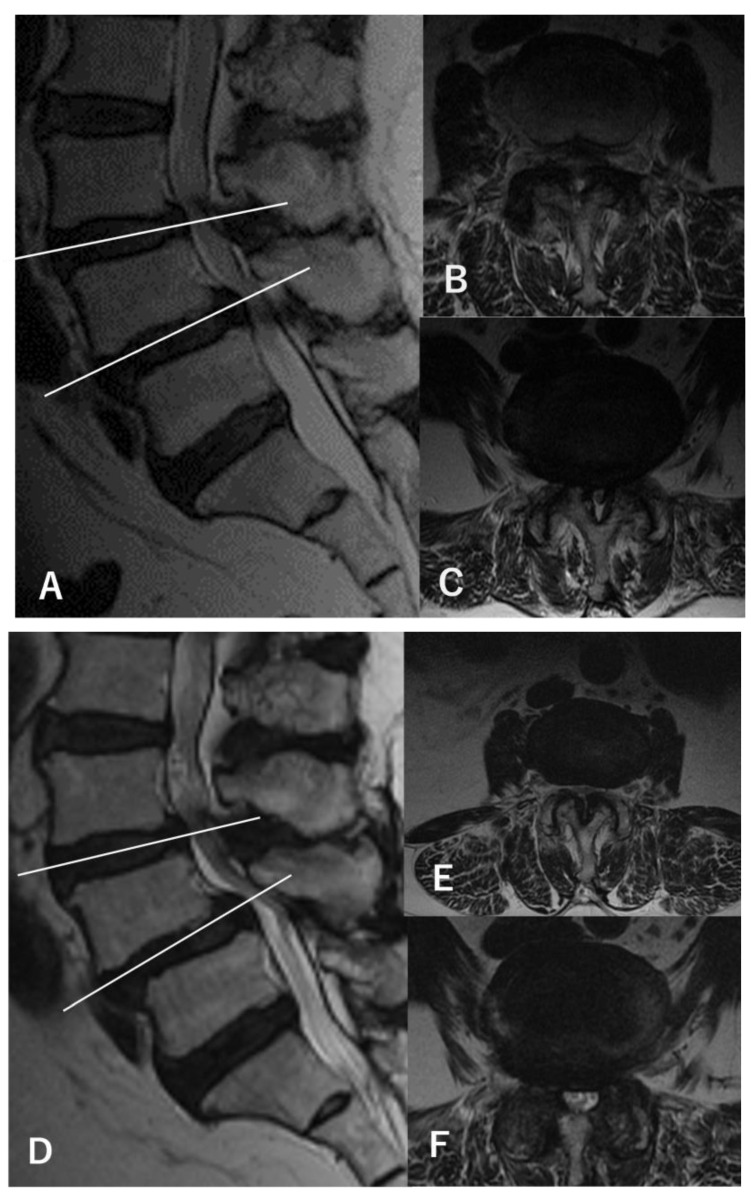
MRI findings of a representative case (a 67-year-old woman). (**A**) Preoperative sagittal-view MRI shows L3–4 and L4–5 stenosis with L4 spondylolisthesis. (**B**) The axial view of L3–4 shows a dural sac cross-sectional area (DSCA) of 42.6 mm^2^. (**C**) The axial view of L4–5 shows a DSCA of 28.3 mm^2^. The patient showed only bilateral L5 radiculopathy without any L4 findings during walking and standing load tests, and L4–5 decompression was performed using a microendoscope (microendoscopic laminectomy; MEL). After surgery, the symptoms disappeared, and no recurrences were observed within 5 years. Postoperative MRI at 5 years (**D**–**F**) shows only decompression at L4–5, with an L3–4 DSCA of 34.1 mm^2^ (**E**) and an L4–5 DSCA of 84.1 mm^2^ (**F**).

During the entry period, 178 patients underwent L4–5-level surgery for LSS, and 6 patients were excluded according to the exclusion criteria. Another 35 patients were excluded because of a lack of preoperative NRS for symptoms or RDQ data. Finally, 137 patients were analyzed in this study (Figure 1). From the evaluation of preoperative MRI, 16 patients were determined to have asymptomatic L3–4 stenosis in addition to symptomatic L4–5 stenosis (double-level stenosis with asymptomatic L3–4 stenosis group: double group) (Figure 2). The remaining 121 patients had only L4–5 stenosis (single-level stenosis group: single group). No stenotic levels other than L3–4 and L4–5 were present in any cases. After surgery, 98 cases (72%) completed the 5-year follow-up.

### 3.2. Characteristics of Patients in the Single and Double Groups

No significant differences were seen between the double and single groups in age, sex, BMI, DM, smoking status, L4 spondylolisthesis, use of microendoscope, NRS scores of symptoms, or RDQ scores for preoperative evaluation (Table 2). The mean L3–4 DSCA in the double group was 43.9 mm^2^, which is significantly smaller than the 100.6 mm^2^ seen in the single group (*p* < 0.05). No significant differences in preoperative assessment were evident between patients with or without 5-year follow-up (Supplementary Table S1).

### 3.3. Outcomes of 5-Year Follow-up

During the 5-year follow-up period, two patients in the double group (16.7%) and nine patients in the single group (10.5%) presented with a new onset of symptoms originating from L3–4, showing no significant difference in the incidence of new-onset L4 symptoms between groups (Table 3). Additional surgeries for L3–4 stenosis were performed for only one patient in the double group (8.3%) and three patients in the single group (3.5%), again showing no significant differences between groups (Table 3). L5 residual symptoms were observed in 6 patients in the double group (50%) and 38 patients in the single group (44.2%) (Table 3). Revision surgery for L4–5 was performed for one patient in the double group (8.3%) and four patients in the single group (4.7%). No significant differences were seen in the incidence of L5 residual symptoms or the revision surgery rate (Table 3). Comparing pre- and postoperative symptoms and RDQ results, the single group showed significant improvements in all symptoms for the NRS scores and RDQ scores (*p* < 0.05), whereas the double group only showed improvements in the NRS score for leg pain (*p* < 0.05) (Table 4). Evaluating changes in the NRS and RDQ scores before and after surgery, no significant differences were identified in changes to the scores for all symptoms or RDQ scores between groups (Table 4).

## 4. Discussion

This study showed that asymptomatic L3–4 stenosis evident on preoperative MRI did not consistently induce symptoms within 5 years of surgery. Incidences of L4 symptoms and additional surgery for the L3–4 level were unrelated to preoperative MRI findings. These results indicate that excluding asymptomatic stenosis from surgical areas diagnosed based on neurological findings is reasonable, even when severe stenosis is apparent on MRI. All symptoms improved in both groups, but significant improvement was only observed for leg pain in the double group; meanwhile, all symptoms improved significantly in the single group. On the other hand, no significant differences in changes to symptoms over 5 years or incidences of residual symptoms were seen between groups. Therefore, L4–5 decompression was considered effective for at least 5 years in both groups.

In the diagnosis of LSS, MRI is the most valuable imaging modality to visualize the lumbar spinal canal without invasive procedures. A DSCA under 75–100 mm^2^ was considered to represent stenosis in an in vitro and clinical study [22]. One previous report showed a significant correlation between walking distance and cross-sectional area of the most severely stenosed level in LSS patients [23]. In terms of the relationship between MRI findings and response to conservative therapy, a smaller DSCA is related to the risk of failure for conservative treatment [24]. Another study using myelography showed that conservative or operative treatment was more effective for patients with moderate stenosis than for those with severe stenosis [25]. In terms of surgical outcomes, Mannion et al. reported that severe stenosis correlated with poor surgical outcomes [26], while Moojen et al. showed that the degree of stenosis on MRI did not correlate with disability or prognosis [27]. These findings indicated that the degree of stenosis is associated with both disease severity and treatment outcome. However, they only suggest that symptomatic stenosis correlates with the degree of stenosis, and does not refer to the level of asymptomatic stenosis in symptomatic patients. Many studies have conversely reported finding no correlations between DSCA and severity of symptoms [7,28], quality-of-life measures [8,9,28], or walking distance [10] among LSS patients. Thus, the relationship between DSCA and symptoms is not as simple as “severe stenosis indicates severe disease”. Further, asymptomatic stenosis on MRI is often seen in the elderly [4,5,6]. One Japanese epidemiological study showed that 64% (98/153) of individuals with a DSCA < 50 mm^2^ showed LSS symptoms, but this value was not predictive of newly progressed LSS symptoms after 1 year [6]. Another study showed that about 30% of participants had severe central stenosis on MRI, whereas only 17.5% were symptomatic [29]. These findings indicated that severe stenosis does not always induce symptoms. However, most patients (87–96%) who undergo surgery show a DSCA lower than 70 mm^2^ [9,28], so a low DSCA is related to indications for surgery. Aaen et al. examined other MRI findings, such as disc degeneration, facet tropism, and fatty infiltration of paraspinal muscles in addition to DSCA, but found no associations with symptoms [28]. Other factors in addition to axial stenosis must be associated with the presence of symptomatic pathology.

MRI is essential for reaching a diagnosis in clinical practice, but LSS should not be diagnosed solely from findings of anatomical stenosis on MRI. Our diagnostic criteria, including the gait-loading test and lumbar extension-loading test, can distinguish asymptomatic stenosis from symptomatic stenosis, and seem helpful for determining the minimum number of levels to decompress in cases with multiple-level stenosis [11,14]. In terms of the natural course of asymptomatic stenosis, Tsutsumimoto et al. observed asymptomatic lumbar spinal stenosis in patients undergoing cervical surgery [30]. They found that 82.1% of patients (32 of 39) with asymptomatic stenosis did not develop lumbar-related leg symptoms within 5 years after surgery. They concluded that prophylactic lumbar decompression is unnecessary for concomitant asymptomatic lumbar spinal stenosis in patients with cervical myelopathy. The present study investigated patients with LSS and asymptomatic stenosis for the same period of time, and considered that prophylactic decompression is unnecessary for asymptomatic levels. Minimizing the number of decompression levels as much as possible is thought to contribute to reducing the invasiveness of surgery, leading to fewer complications and better surgical outcomes [31]. On the other hand, it was unclear in our cases whether decompression of asymptomatic stenosis resulted in any difference in outcomes. One basic study showed that double-level nerve root compression had a greater effect on neurological function than single-level compression [32]. Theoretically, symptoms are more likely to appear if double stenosis is present [33]. Thus, asymptomatic L3–4 stenosis may have some effect, even in the absence of symptoms. The present study showed that asymptomatic L3–4 stenosis did not affect the severity of clinical symptoms of L4–5 stenosis nor the outcomes of L4–5 decompression. Further long-term follow-up is necessary to clarify the influence of asymptomatic stenosis on the clinical situation.

Several limitations to this study must be considered. Firstly, the sample size was small. The double group included only 16 cases, with a 5-year follow-up rate of about 70%. This may have been insufficient to detect significant differences. However, the incidences of new symptoms and the need for additional surgery were both considered low enough to draw tentative conclusions. No significant differences were identified between patients with and without 5 years of follow-up, so selection bias due to dropout was not considered problematic. Secondly, changes in the definitions of stenosis may have changed some parts of the results. However, the finding that patients with severe asymptomatic stenosis remained free of symptoms for 5 years was unaffected by how stenosis was defined. We will examine suitable cut-off points for the DSCA in another study. Thirdly, the follow-up period for this study was only 5 years. This may have been too short to thoroughly clarify the future of asymptomatic stenosis. Thus, we aim to continue follow-up with patients, such as for 10 years or longer. Fourthly, this study only examined patients with L4–5 stenoses. Whether patients with multiple symptomatic stenoses would show similar results is not clear. Fifthly, some doubt remains as to whether limited decompression for symptomatic stenosis is superior to non-selective decompression for multiple stenotic levels on MRI. Although we consider only decompression for symptomatic levels to be less invasive, a randomized controlled trial with longer follow-up is needed to address this question.

## 5. Conclusions

This study observed the natural course of asymptomatic untreated L3–4 stenosis after L4–5 decompression. Asymptomatic stenosis became symptomatic within 5 years in only two patients. No significant differences in 5-year surgical outcomes for L4–5 decompressions were identified in patients with well-differentiated L4–5 stenosis showing asymptomatic L3–4 stenosis compared to patients with L4–5 stenosis without image-based findings of L3–4 stenosis.

## Figures and Tables

**Table 1:** Diagnostic criteria for symptomatic level and neurological findings.

	Motor	Sensory	Reflex
L4	Quadriceps	Medial knee	PTR
L5	TA EHL EDL	Lateral lower extremity and dorsal foot	
S1	Gastrocnemius FHL FDL	Lateral foot	ATR

TA: tibialis anterior; EHL: extensor hallucis longus; EDL: extensor digitorum longus; FHL: flexor hallucis longus; FDL: flexor digitorum longus; PTR: patellar tendon reflex; ATR: Achilles tendon reflex.

**Table 2:** Preoperative comparison between cases with or without asymptomatic stenosis.

	Double Group Mean [SD], *n* (%)	Single Group	*p*-Value
Cases	16	121	
Age (y)	68.6 [9.3]	66.7 [10.3]	0.45
Sex Male Female	10 (62.5) 6 (37.5)	73 (60.3) 48 (39.7)	0.87
BMI	25.9 [2.1]	24.9 [3.3]	0.23
Smoking No Past Present	11 (68.9) 4 (25) 1(6.3)	78 (64.5) 23 (19) 20 (1.5)	0.53
DM	4 (25)	18 (14.9)	0.26
L3–4 DSCA (mm^2^)	43.9 [4.5]	100.6 [38.7]	<0.01
L4 spondylolisthesis	5(31.3)	56(46.3)	0.26
MEL	4 (25)	31(25.6)	0.67
NRS scores			
Low back pain	5.8 [3.3]	3.8 [3.3]	0.06
Leg pain	5.2 [3.3]	5.5 [3.6]	0.72
Leg numbness	6.1 [3.7]	5.8 [3.1]	0.67
RDQ total score	10.6 [6.0]	11.2 [5.7]	0.72
RDQ deviation score	38.3 [8.7]	39.1 [10.1]	0.86

DM: diabetes mellitus; DSCA: dural sac cross-sectional area; MEL: microendoscopic laminectomy; NRS: numerical rating scale; RDQ: Roland–Morris Disability Questionnaire.

**Table 3:** Incidences of symptoms and surgery 5 years after surgery.

	Double Group *n* (%)	Single Group	*p*-Value
Cases	12	86	
L4 symptoms	2 (16.7)	9 (10.5)	0.54
L3–4 surgery	1 (8.3)	3 (3.5)	0.47
Residual symptoms	6 (50)	38 (44.2)	0.7
L4–5 revision surgery	1 (8.3)	4 (4.7)	0.61

**Table 4:** Comparison between results preoperatively and at 5-year follow-up examination.

	Double Group (n = 10) Mean [SD]				Single Group (n = 63)				*p*-Value #
	Pre-Op	5-year Post-Op	Post-op Change	*p*-Value *	Pre-op	5-year Post-Op	Post-op Change	*p*-Value *	
NRS scores									
Low back pain	5.1 [3.4]	4.1 [2.9]	−0.9 [3.2]	0.41	3.4 [3.3]	2.5 [3.2]	−1.0 [3.5]	0.03	0.97
Leg pain	5.2 [3.4]	2.5 [2.8]	−2.7 [3.6]	0.04	5.2 [3.4]	2.5 [2.9]	−2.7 [3.7]	<0.01	0.99
Leg numbness	5.7 [4.0]	3.2 [2.4]	−2.4 [4.9]	0.18	5.3 [3.2]	3.2 [3.3]	−2.2 [3.9]	<0.01	0.84
RDQ scores									
Total score	11.9 [5.7]	8.6 [6.3]	−3.3 [5.3]	0.15	11.4 [5.5]	5.7 [5.8]	−5.7 [7.4]	<0.01	0.4
Norm-based score	36.5 [9.6]	43.0 [11.3]	6.5 [10.1]	0.14	39.8 [9.7]	49.4 [10.9]	9.6 [13.8]	<0.01	0.57

* Comparison between preoperative and 5-year postoperative scores. # Comparison of postoperative changes in scores between asymptomatic and single groups. NRS: numerical rating scale; RDQ: Roland–Morris Disability Questionnaire.

## Data Availability

The datasets generated and analyzed during the current study are not publicly available, but are available from the corresponding author on reasonable request.

## Supplementary Materials

The following supporting information can be downloaded at: https://www.mdpi.com/article/10.3390/medicina60040636/s1, Table S1: Preoperative comparison between cases with or without follow-up for 5 years after surgery.
