# Supplementary material for: Time Course of Asymptomatic Stenosis in Multiple Lumbar Spinal Stenosis—Five-Year Results of Selective Decompression of Symptomatic Levels
Source: Medicina (Kaunas). 2024 Apr 15;60(4):636. doi: 10.3390/medicina60040636 (PMC11052377; doi:10.3390/medicina60040636)
Supplement: Supplementary file 1 [file medicina-60-00636-s001.zip › medicina-2912728-supplementary.pdf]

medicina MDPI

Supplementary Table S1. Preoperative comparison between cases with or without follow-up for 5 years after surgery

	Follow-up (+) Mean [SD], n (%)	Follow-up (-)	P-value
Cases	98	39	
Age (y)	66.8 [8.8]	67.9 [11.7]	0.42
Sex			0.81
Male	60 (61.2)	23 (59)	
Female	38 (38.8)	16 (41)	
BMI (kg/m^2)	25.0 [2.9]	25.7 [4.2]	0.15
Smoking			0.32
No	63 (64.3)	26 (66.7)	
Past	22 (22.5)	5 (12.8)	
Present	13 (13,3)	8 (20.5)	
DM	15 (15.3)	7 (18.0)	0.70
L3–4 DSCA	89.6 [37.7]	104.7 [46.2]	0.07
L3–4 stenosis	12 (12)	4 (10)	0.74
L4 spondylolisthesis	40 (40.8)	21 (53.9)	0.17
MEL	22 (22.5)	13(33.3)	0.19
NRS scores			
Low back pain	3.9 [3.3]	4.2 [3.4]	0.66
Leg pain	5.4 [3.2]	4.7 [3.6]	0.46
Leg numbness	5.6 [3.2]	6.5 [2.8]	0.17
RDQ total score	11.3 [5.8]	10.5 [5.1]	0.80
RDQ deviation score	39.2 [10.2]	39.2 [8.7]	0.88

DM: diabetes mellitus; DSCA: dural sac cross-sectional area; NRS: numerical rating scale; RDQ: Roland–Morris Disability Questionnaire; SD: standard deviation.
